# Supplementary material for: The unifying catalytic mechanism of the RING-between-RING E3 ubiquitin ligase family
Source: Nat Commun. 2023 Jan 11;14:168. doi: 10.1038/s41467-023-35871-z (PMC9834252; doi:10.1038/s41467-023-35871-z)
Supplement: Supplementary file 1 — Supplementary Information [file 41467_2023_35871_MOESM1_ESM.pdf]

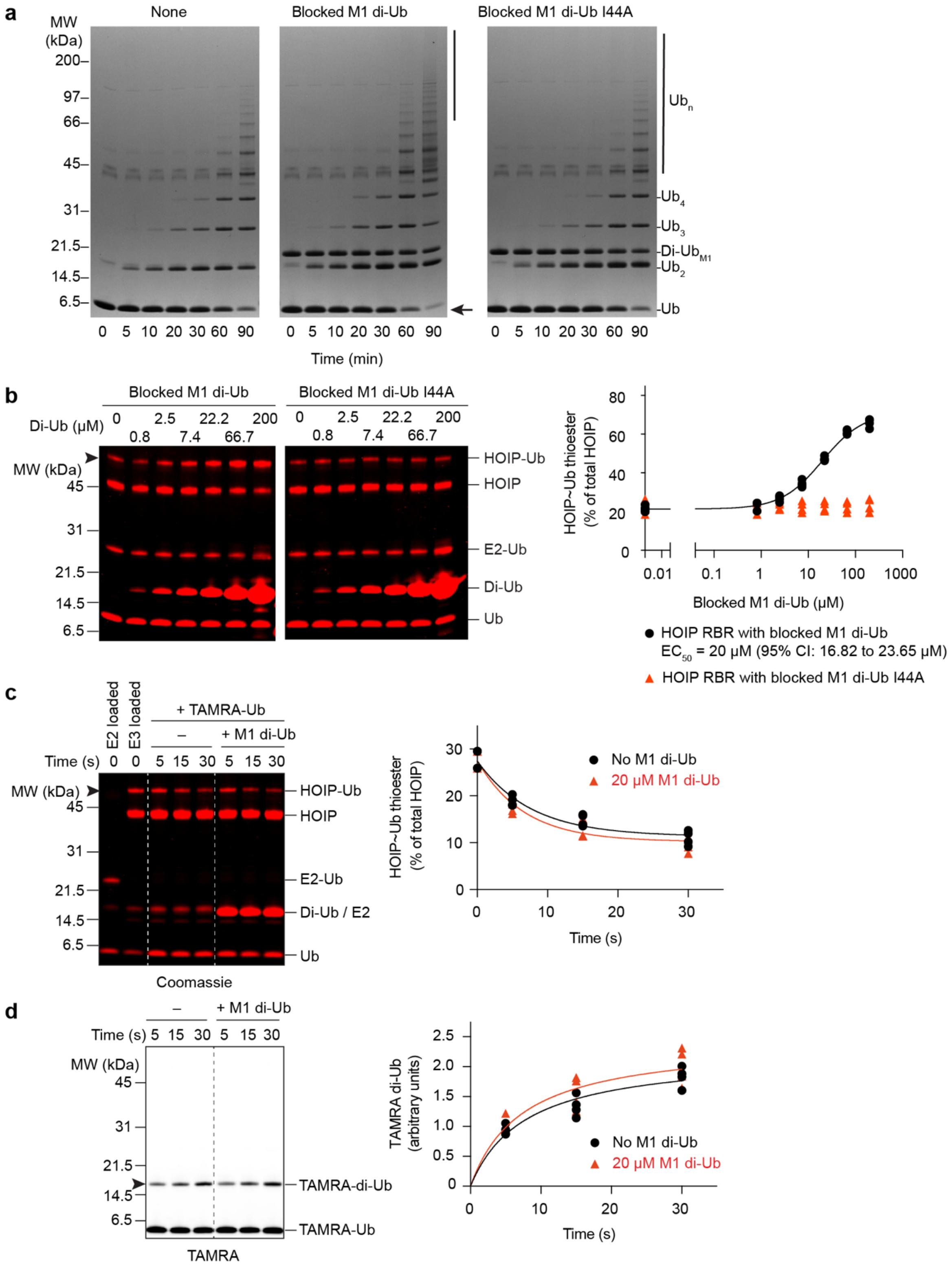

Supplementary Figure 1: Allosteric Ub does not activate the aminolysis reaction in HOIP
a M1-linked Ub chain formation assay probing both steps of the HOIP RBR E3 ligase reaction. Blocked M1-linked di-Ub, but not I44A mutant blocked M1-linked di-Ub, accelerates formation of long Ub chains (Ub$_n$, black bar) and depletion of mono-Ub (arrow). Blocked di-Ub contains an N-terminal Gly-Pro-Gly (GPG) extension and has the C-terminal Gly deleted, preventing participation in the catalytic reaction as a donor or acceptor Ub, while still allowing binding to the allosteric site. Representative gels of three independent experiments performed under slightly different conditions with consistent results are shown. **b** HOIP-Ub thioester formation assay probing the first (transthiolation) step of the HOIP RBR E3 ligase reaction. HOIP-Ub thioester was generated for 30 sec at 25°C. Left: Coomassie-stained gels. The arrowhead indicates HOIP-Ub bands used for analysis. Right: Quantification of the HOIP-Ub band normalized to total HOIP (HOIP + HOIP-Ub). (n = 4, individual data points and representative gels shown). **c/d** HOIP-Ub transfer assay probing the second (aminolysis) step of the HOIP

RBR E3 ligase reaction. HOIP was pre-loaded with N-terminally blocked Ub (GPG-Ub) and the Ub transfer initiated by addition of TAMRA-Ub substrate. HOIP-Ub discharge was followed using Coomassie-stained SDS-PAGE (**c**, arrowhead indicates HOIP-Ub bands used for analysis), whereas TAMRA-di-Ub formation was followed using the TAMRA-fluorescence signal (**d**, arrowhead indicates TAMRA-di-Ub bands used for analysis). HOIP-Ub discharge and TAMRA-di-Ub formation were quantified and plotted. (n = 4, individual data points and representative gels shown). Source data are provided as a Source Data file.

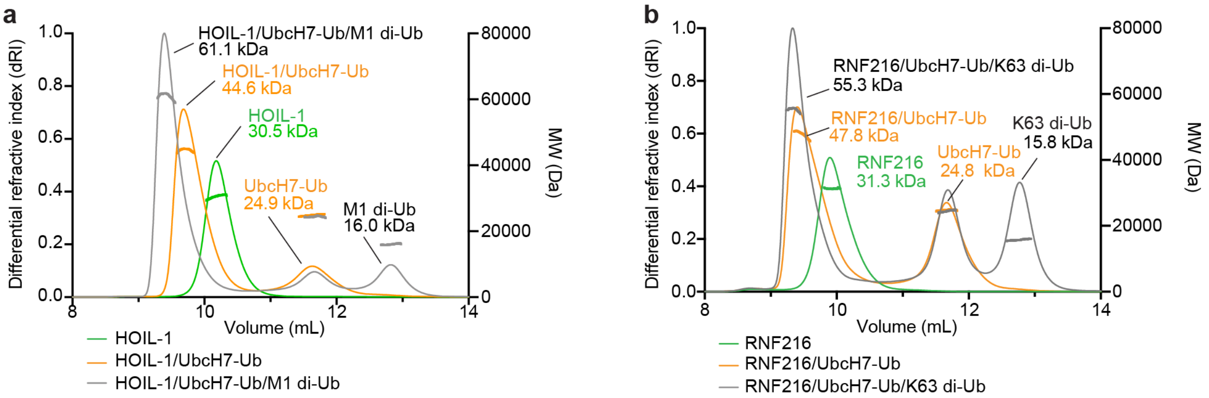

c

Protein	Calculated MW (kDa)	Observed MW (kDa)	Discrepancy (kDa)
HOIL-1 helix-RBR (C460A)	31.7	30.5	-1.2
HOIL-1 helix-RBR (C460A)/UbcH7-Ub	58.3	44.6	-13.7
HOIL-1 helix-RBR (C460A)/UbcH7-Ub/M1 di-Ub	75.3	61.1	-14.2
UbcH7-Ub	26.6	24.9	-1.7
M1 di-Ub	17	16.0	-1.0
RNF216 RBR-helix (C688A, pSer719)	31.4	31.3	-0.1
RNF216 RBR-helix (C688A, pSer719)/UbcH7-Ub	58	47.8	-10.2
RNF216 RBR-helix (C688A, pSer719)/UbcH7-Ub/K63 di-Ub	75	55.3	-19.7
UbcH7-Ub	26.6	24.8	-1.8
K63 di-Ub	17	15.8	-1.2

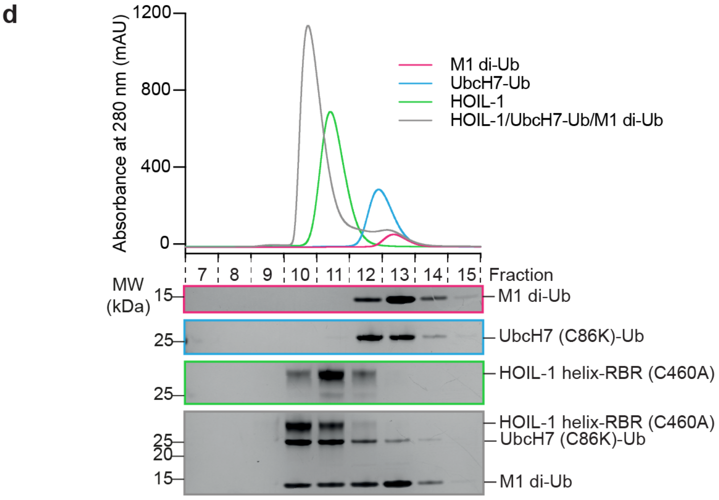

Supplementary Figure 2: RBR/UbcH7-Ub/di-Ub ternary complex formation measured by SEC-MALS.
a HOIL-1helix-RBR (C460A), and complexes of HOIL-1helix-RBR (C460A) with UbcH7(C86K)-Ub or UbcH7(C86K)-Ub and M1-linked di-Ub were analysed by size-exclusion chromatography coupled to multi-angle light scattering (SEC-MALS). HOIL-1 forms a binary complex with the UbcH7-Ub isopeptide conjugate, and a ternary complex with the UbcH7-Ub isopeptide conjugate and M1-linked di-Ub as indicated by an increase in molecular mass and associated shift in elution volume. **b** SEC-MALS analysis of RNF216 RBR-helix (C688A, pS719), and complexes of RNF216 RBR-helix (C688A, pS719) with UbcH7(C86K)-Ub or UbcH7(C86K)-Ub and K63-linked di-Ub. RNF216 forms a binary complex with UbcH7(C86K)-Ub, and a ternary complex with UbcH7(C86K)-Ub and K63-linked di-Ub as indicated by an increase in molecular mass and associated shift in elution volume. **c** Summary of SEC-MALS statistics. **d** SEC chromatogram and SDS-PAGE analysis of fractions for HOIL-1 complex purification. When mixed, HOIL-1 helix-RBR (C460A), UbcH7(C86K)-Ub and M1-linked di-Ub co-elute in the same fractions suggesting ternary complex formation (n = 1). Source data are provided as a Source Data file.

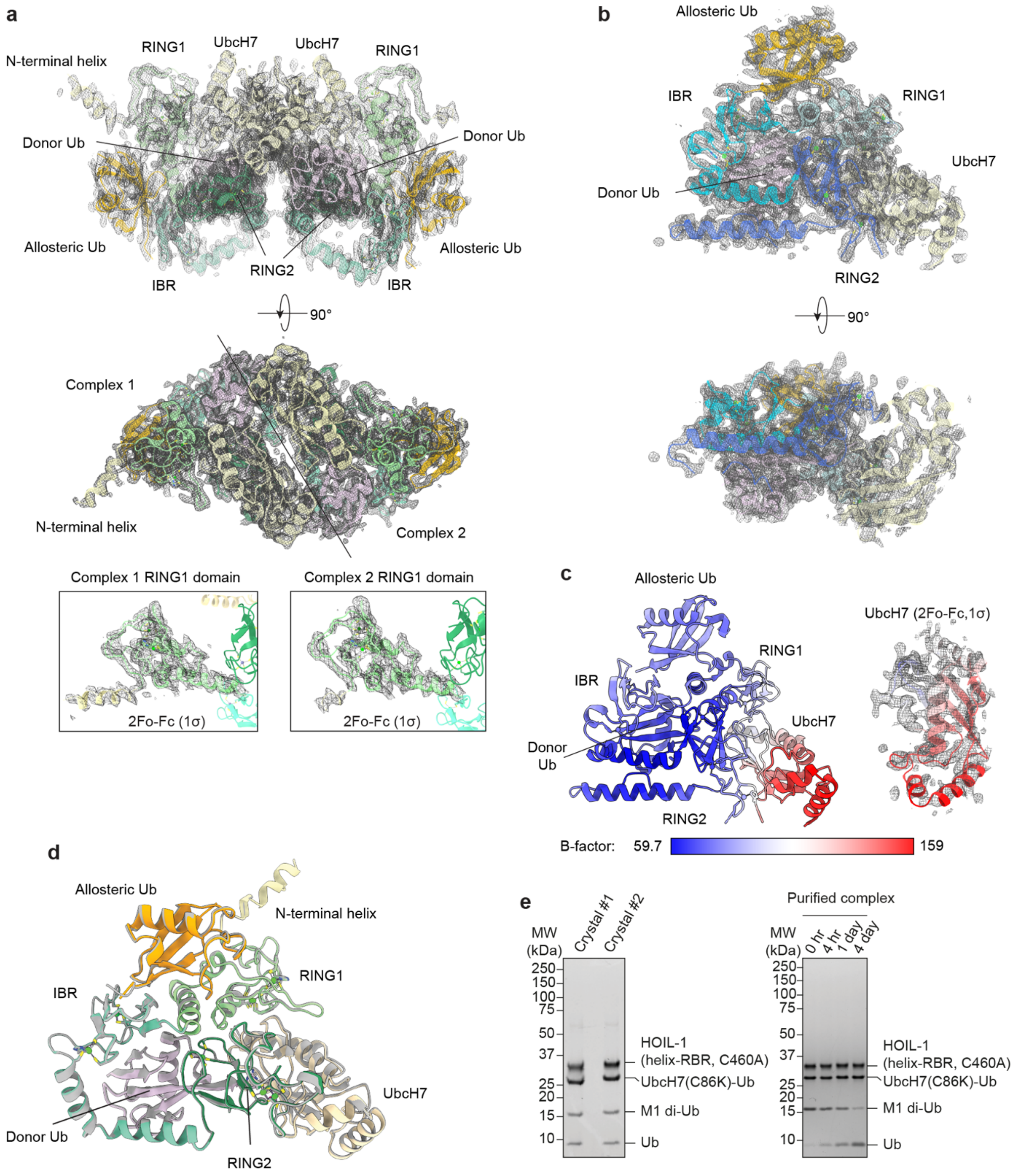

Supplementary Figure 3: Crystal structures of RBR transthiolation complexes
a The asymmetric unit of the HOIL-1 transthiolation complex with 2Fo-Fc electron density map contoured at 1σ. The asymmetric unit contains two copies of the transthiolation complex consisting of HOIL-1 helix-RBR (C460A; green), UbcH7(C86K)-Ub (pale yellow and pink) and allosteric Ub (orange). Density for the N-terminal helix of HOIL-1 helix-RBR is resolved in only one copy of the complex (see insets). **b** The asymmetric unit of the RNF216 transthiolation complex with 2Fo-Fc electron density map contoured at 1σ. The asymmetric unit contains a single copy of the transthiolation complex consisting of RNF216 RBR-helix (C688A, pSer719; blue), UbcH7(C86K)-Ub (pale yellow and pink) and allosteric Ub (orange). **c** RNF216 transthiolation complex coloured by B-factor. The modelled UbcH7 molecule has significantly higher B-factors and weaker electron density than other parts of the complex. **d** Overlay of the two copies of the HOIL-1 complex from the asymmetric unit. The two complexes align with an RMSD of 0.396Å. **e** SDS-PAGE analysis of two harvested HOIL-1 complex crystals (left panel). Incubation of the HOIL-1 transthiolation complex at room temperature for 4 days reveals cleavage of the M1-linked di-Ub and concomitant appearance of mono-Ub (n = 1). Source data are provided as a Source Data file.

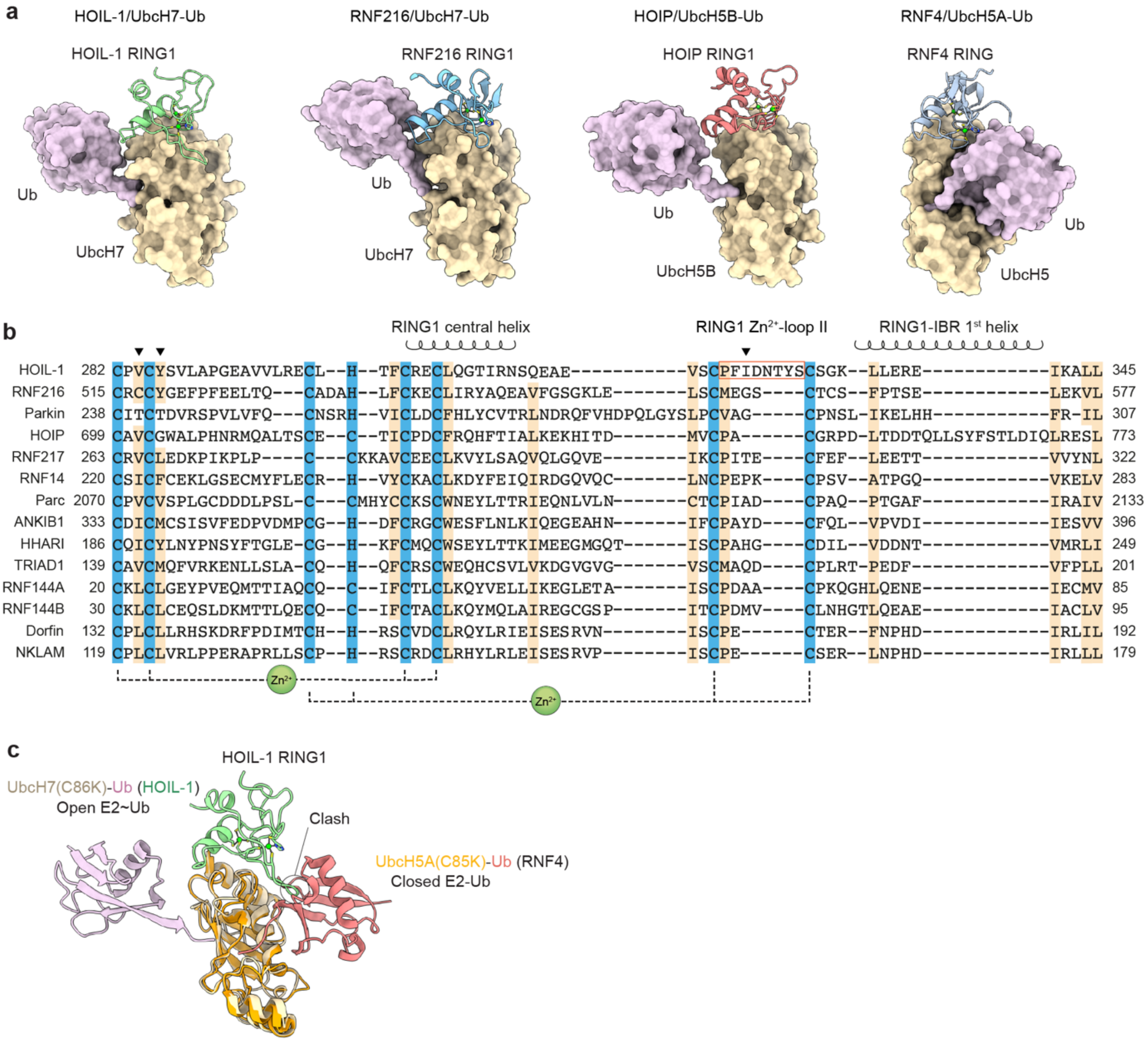

Supplementary Figure 4: RBR RING1 interactions with E2

a Comparison of RBR E3 RING1 and RNF4 E3 RING domains bound to E2-Ub conjugates. RING1 and RING domains bind to the same site of the E2. RBR RING1 domains stabilise an open E2-Ub conformation while the canonical RING stabilises a closed E2-Ub conformation. The E2-Ub bound structures of HOIL-1, RNF216, HOIP (PDB: 5EDV, ref.[1]) and RNF4 (PDB: 4AP4, ref.[2]) are shown from left to right. **b** RING1 sequence alignment of all 14 human RBR family members. Zinc coordinating residues and conserved hydrophobic residues are highlighted in blue and beige, respectively. E2 interacting residues mutated in Fig. 4e,f are denoted by black arrowheads. **c** The HOIL-1 RING1 Zn^{2+}-loop II clashes with E2-Ub in its closed conformation. The HOIL-1/E2-Ub structure (this study) is aligned (by E2) and overlayed with RNF4/E2-Ub structure (PDB: 4AP4, ref.[2]). The RNF4 RING domain is hidden for simplicity. HOIL-1 RING1 Zn^{2+}-loop II clashes with the donor Ub from the RNF4 structure.

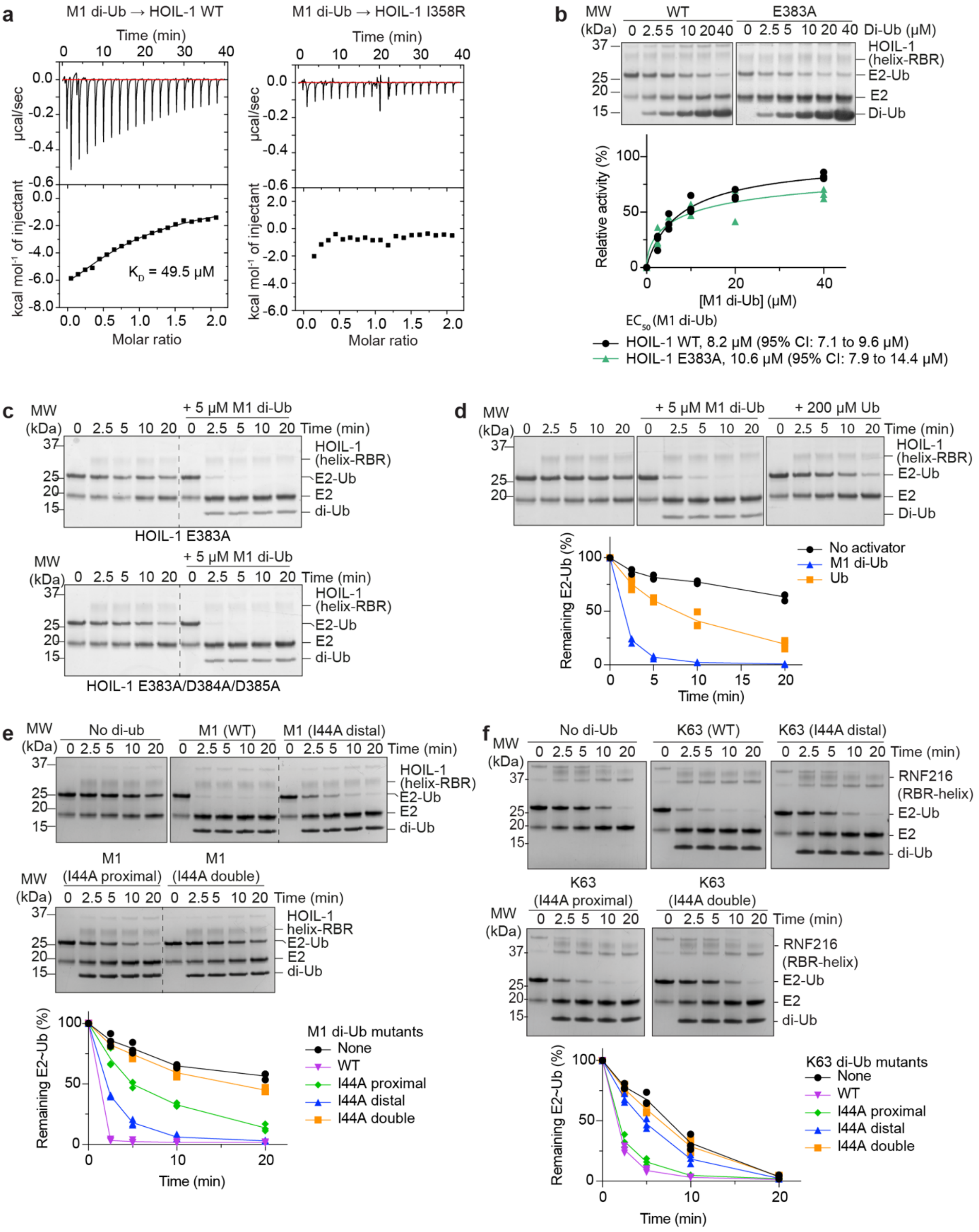

Supplementary Figure 5: Allosteric activation of HOIL-1 and RNF216
a ITC experiments testing binding of M1 di-Ub to HOIL-1 helix-RBR WT and allosteric site mutant I358R. **b** WT and E383A HOIL-1 helix-RBR catalysed UbcH7-Ub discharge assay in the presence of increasing concentrations of M1-linked di-Ub. The bottom panel shows quantification of three independent experiments for EC_{50} determination. Nonlinear regression curves were fitted using the [agonist] vs. normalised responses – variable slope model. **c** Time-course of HOIL-1 helix-RBR catalysed UbcH7-Ub discharge assay comparing HOIL-1 E383A and the E383A/D384A/D385A triple mutant in the absence and presence of 5 µM M1 di-Ub. Representative gels of three independent experiments are shown. **d** Time-course of HOIL-1 helix-RBR catalysed UbcH7-Ub discharge assay without and with allosteric activators M1-linked di-Ub and mono-Ub, showing that mono-Ub can activate HOIL-1 at a very high concentration. The top panels show representative SDS-PAGE gels.

The bottom shows quantification of three independent experiments. **e** Time-course of the HOIL-1 helix-RBR catalysed UbcH7-Ub discharge assay in the presence of different M1 di-Ub mutants. Quantification of three independent experiments is shown at the bottom. **f** Time-course of the RNF216 RBR-helix catalysed UbcH7-Ub discharge assay in the presence of different K63 di-Ub mutants. Quantification of three independent experiments is shown at the bottom. Source data are provided as a Source Data file.

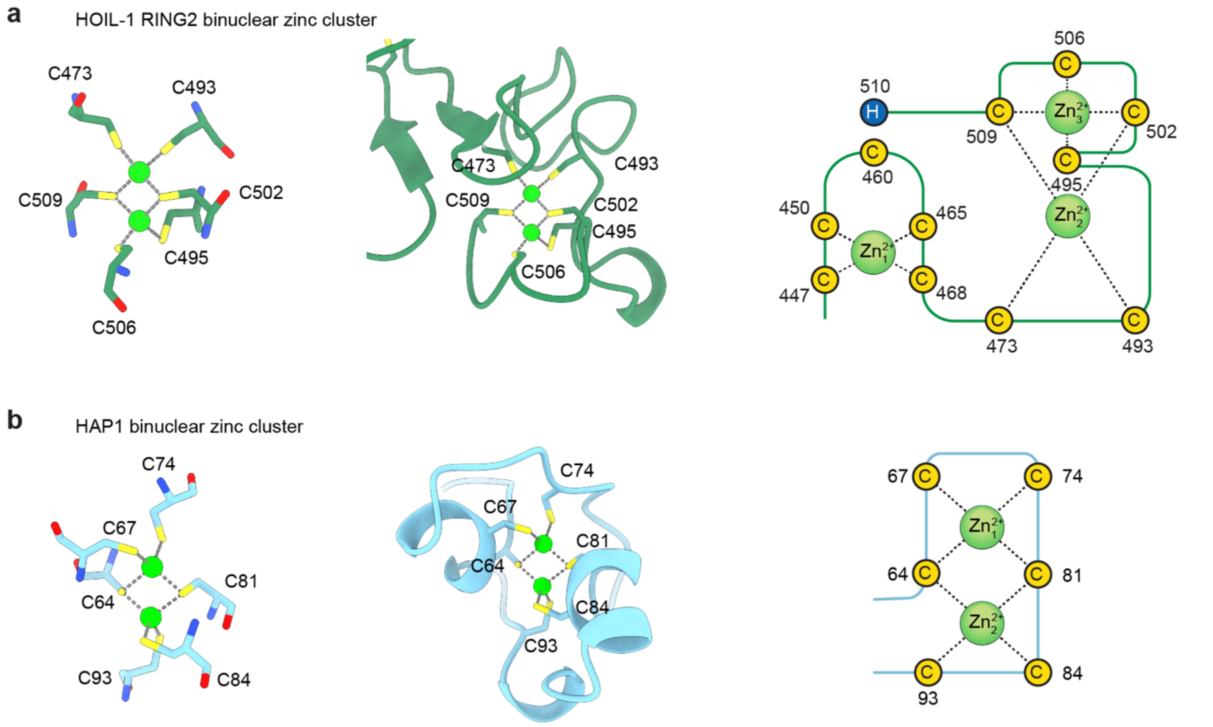

Supplementary Figure 6: Comparison of the HOIL-1 and Zn2/Cys6 binuclear clusters of yeast transcription factors
a Structure of the HOIL-1 RING2 Zn2/Cys6 binuclear cluster with zinc coordinating residues shown in stick representation and zinc as green spheres. The topology of zinc coordinating residues is shown in the right panel.
b Structure of the Zn2/Cys6 binuclear cluster of the *S. cerevisiae* GAL4-family HAP1 transcription factor (PDB 2HAP, ref.[3]) with zinc coordinating residues shown in stick representation and zinc as green spheres. The topology of zinc coordinating residues is shown in the right panel.

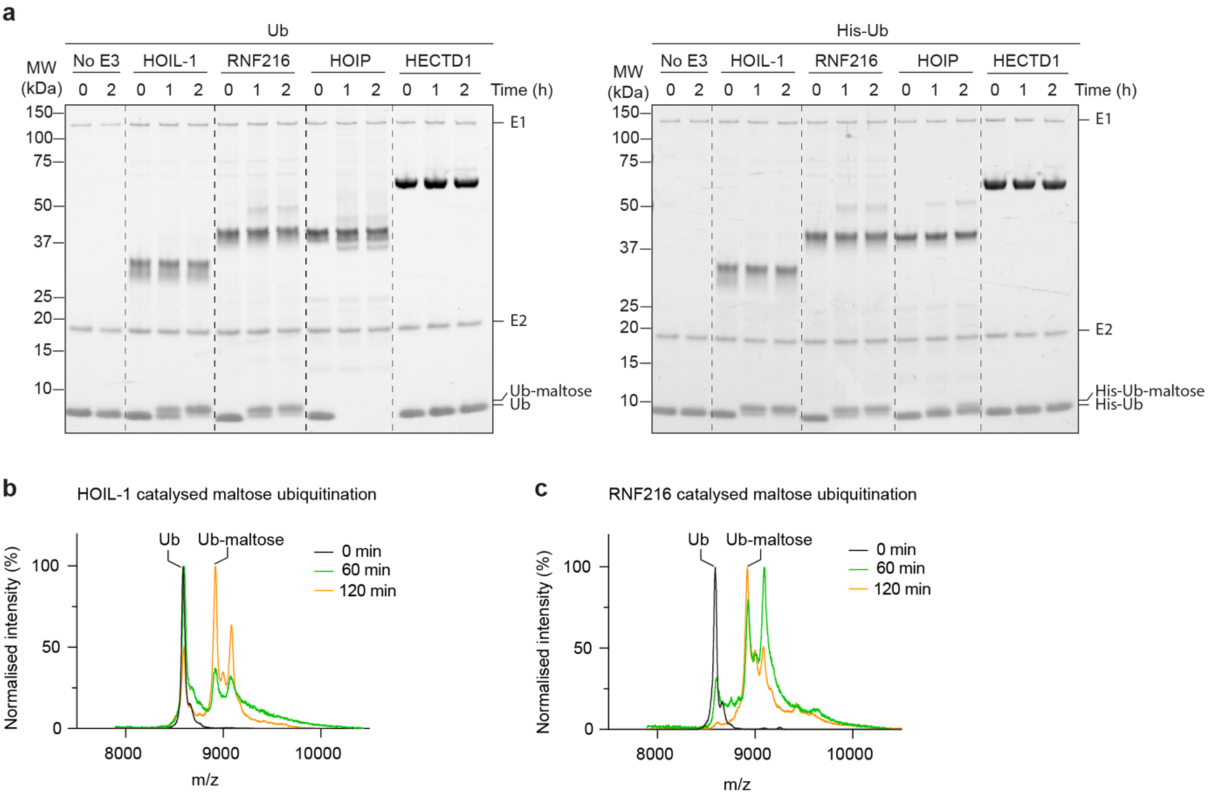

Supplementary Figure 7: Maltose ubiquitination by HOIL-1, RNF216 and other E3 ligases
a Time-course of the maltose ubiquitination assay using native Ub (left panel) and His-Ub (right panel). HOIL-1 and RNF216 catalyse Ub-maltose formation in both cases. HOIP readily synthesises Ub chains when native Ub is used but weakly synthesises His-Ub-maltose with His-Ub that cannot form M1-linked Ub chains. HECTD1 auto-ubiquitinates (red arrowhead) but does not ubiquitinate maltose. Representative gels of three independent experiments performed under slightly different conditions yielding consistent results are shown. **b** MALDI-TOF spectra of HOIL-1 catalysed maltose ubiquitination (n = 1). Baseline is subtracted from individual spectra, and peaks are normalised to the largest value within the displayed range for each reaction time. m/z, mass-to-charge ratio. **c** MALDI-TOF spectra of RNF216 catalysed maltose ubiquitination (n = 1), processed as in panel b. Source data are provided as a Source Data file.

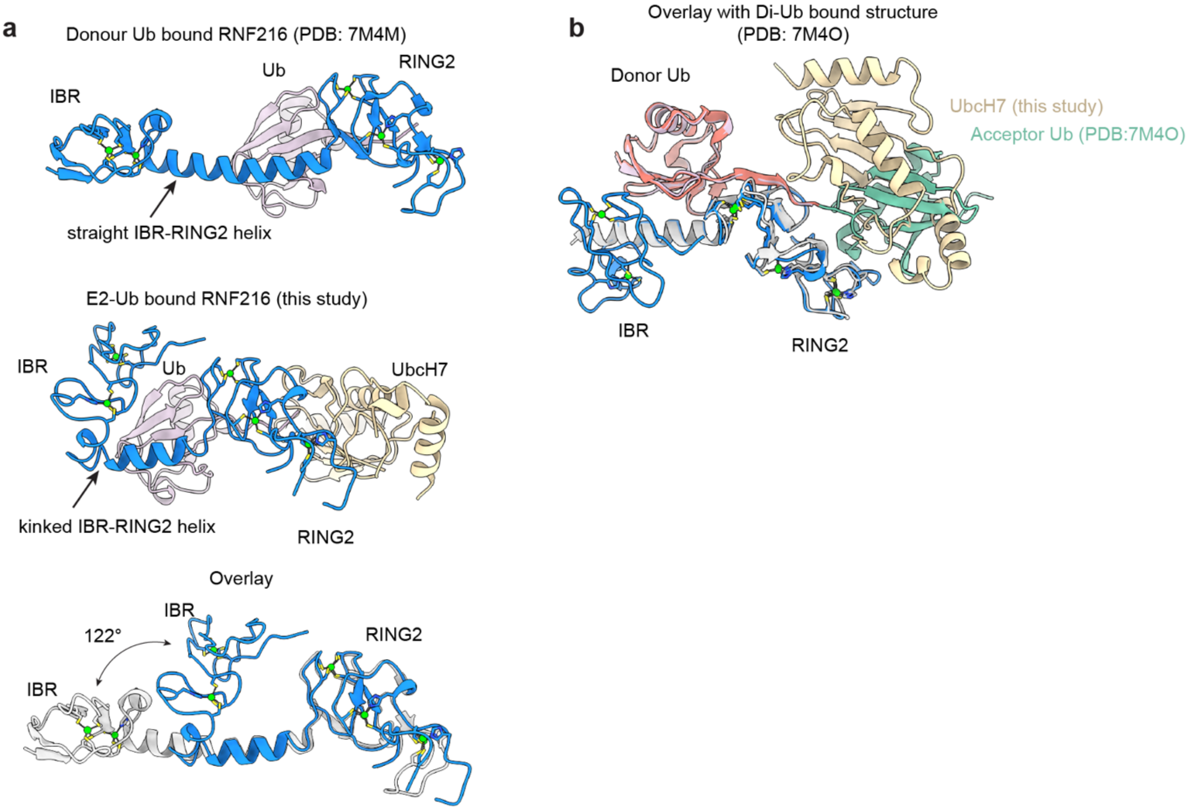

Supplementary Figure 8: RNF216 conformational dynamics and E2 and acceptor Ub binding
a Comparison of RNF216 IBR-RING2 structures from Ub bound (top panel, PDB: 7M4M, ref.[4]) and E2-Ub bound (middle panel, this study) complexes reveals a significant rearrangement of the IBR-RING2 helix and repositioning of the IBR domain (overlaid in bottom panel). **b** Overlay of K63 di-Ub bound (PDB: 7M4O, ref.[4]) and E2-Ub bound (this study) RNF216 structures show that the E2 and acceptor Ub share an overlapping binding site in the RING2 domain, which would prevent simultaneous binding of E2 and acceptor Ub.

Supplementary References

1. Lechtenberg BC*, et al.* Structure of a HOIP/E2~ubiquitin complex reveals RBR E3 ligase mechanism and regulation. *Nature* **529**, 546-550 (2016).

2. Plechanovova A, Jaffray EG, Tatham MH, Naismith JH, Hay RT. Structure of a RING E3 ligase and ubiquitin-loaded E2 primed for catalysis. *Nature* **489**, 115-120 (2012).

3. King DA, Zhang L, Guarente L, Marmorstein R. Structure of HAP1-18-DNA implicates direct allosteric effect of protein-DNA interactions on transcriptional activation. *Nat Struct Biol* **6**, 22-27 (1999).

4. Cotton TR, Cobbold SA, Bernardini JP, Richardson LW, Wang XS, Lechtenberg BC. Structural basis of K63-ubiquitin chain formation by the Gordon-Holmes syndrome RBR E3 ubiquitin ligase RNF216. *Molecular cell* **82**, 598-615 (2022).
