# Supplementary material for: The unifying catalytic mechanism of the RING-between-RING E3 ubiquitin ligase family
Source: Nat Commun. 2023 Jan 11;14:168. doi: 10.1038/s41467-023-35871-z (PMC9834252; doi:10.1038/s41467-023-35871-z)
Supplement: Supplementary file 4 — Reporting Summary [file 41467_2023_35871_MOESM4_ESM.pdf]

nature portfolio

Corresponding author(s): Bernhard C. Lechtenberg

Last updated by author(s): Dec 17, 2022

Reporting Summary

Nature Portfolio wishes to improve the reproducibility of the work that we publish. This form provides structure for consistency and transparency in reporting. For further information on Nature Portfolio policies, see our Editorial Policies and the Editorial Policy Checklist.

Statistics

For all statistical analyses, confirm that the following items are present in the figure legend, table legend, main text, or Methods section.

n/a	Confirmed	
☐	☒	The exact sample size (*n*) for each experimental group/condition, given as a discrete number and unit of measurement
☐	☒	A statement on whether measurements were taken from distinct samples or whether the same sample was measured repeatedly
☐	☒	The statistical test(s) used AND whether they are one- or two-sided<br>*Only common tests should be described solely by name; describe more complex techniques in the Methods section.*
☒	☐	A description of all covariates tested
☐	☒	A description of any assumptions or corrections, such as tests of normality and adjustment for multiple comparisons
☐	☒	A full description of the statistical parameters including central tendency (e.g. means) or other basic estimates (e.g. regression coefficient) AND variation (e.g. standard deviation) or associated estimates of uncertainty (e.g. confidence intervals)
☐	☒	For null hypothesis testing, the test statistic (e.g. *F*, *t*, *r*) with confidence intervals, effect sizes, degrees of freedom and *P* value noted<br>*Give P values as exact values whenever suitable.*
☒	☐	For Bayesian analysis, information on the choice of priors and Markov chain Monte Carlo settings
☒	☐	For hierarchical and complex designs, identification of the appropriate level for tests and full reporting of outcomes
☒	☐	Estimates of effect sizes (e.g. Cohen's *d*, Pearson's *r*), indicating how they were calculated

Our web collection on statistics for biologists contains articles on many of the points above.

Software and code

Policy information about availability of computer code

Data collection	ASTRA (7.3.1.9, Wyatt Technology), Multi Gauge (Fujifilm), ImageStudio 5.2.5 (Li-COR Biosciences), Image Lab 6.1 (Bio-Rad), Unicorn 7.5 (Cytiva), ITC 200 (Version 1.26.1, GE Healthcare), flexAnalysis 3.4 (Bruker)
Data analysis	XDS (version Nov 1, 2016 and version Feb 5, 2021), Aimless (version 0.7.4), CCP4i2 (version 7.1.016), Phaser (version 2.8.3), Coot (version 0.9.6), Phenix (version 1.19.2_4158 and 1.20.1_4487), Molprobity server (version 4.5.2), CheckMyMetal server (version number not available), PDB Validation Server (validation pipeline 2.29 and 2.30), UCSF Chimera X (versions 1.3 and 1.4), ISOLDE (version 1.3), Origin 7 SR4 (Microcal), ASTRA (7.3.1.9, Wyatt Technology), ImageStudio 5.2.5 (Li-Cor Biosciences), Image Lab 6.1 (Bio-Rad), Prism 9 (GraphPad), Unicorn 7.5 (Cytiva)

For manuscripts utilizing custom algorithms or software that are central to the research but not yet described in published literature, software must be made available to editors and reviewers. We strongly encourage code deposition in a community repository (e.g. GitHub). See the Nature Portfolio guidelines for submitting code & software for further information.

Data

Policy information about availability of data

All manuscripts must include a data availability statement. This statement should provide the following information, where applicable:

- Accession codes, unique identifiers, or web links for publicly available datasets
- A description of any restrictions on data availability
- For clinical datasets or third party data, please ensure that the statement adheres to our policy

The study made use of the following publicly available data sets: PDB entries: 1UBQ [http://doi.org/10.2210/pdb1UBQ/pdb], 2HAP [http://doi.org/10.2210/

pdb2HAP/pdb], 4AP4 [http://doi.org/10.2210/pdb4AP4/pdb], 4BM9 [http://doi.org/10.2210/pdb1UBQ/pdb], 4Q5E [http://doi.org/10.2210/pdb4Q5E/pdb], 5EDV [http://doi.org/10.2210/pdb5EDV/pdb], 6DJW [http://doi.org/10.2210/pdb6DJW/pdb], 7B5L [http://doi.org/10.2210/pdb7B5L/pdb], 7M4M [http://doi.org/10.2210/pdb7M4M/pdb], 7M4O [http://doi.org/10.2210/pdb7M4O/pdb] and 7V8F [http://doi.org/10.2210/pdb7V8F/pdb]; AlphaFold DB models Q9BYM8 [https://alphafold.ebi.ac.uk/entry/Q9BYM8] (HOIL-1) and F8WDI8 [https://alphafold.ebi.ac.uk/entry/F8WDI8] (RNF216). Atomic structures and diffraction data generated in this study have been deposited in the PDB under accession codes 8EAZ (HOIL-1(C460A)/UbcH7(C86K)-Ub/Ub) and 8EB0 (RNF216(C688A)/UbcH7(C86K)-Ub/Ub). All other data supporting the conclusions are available in the article. Biochemical data generated in this study are provided in the Source Data file.

Human research participants

Policy information about studies involving human research participants and Sex and Gender in Research.

Reporting on sex and gender	N/A
Population characteristics	N/A
Recruitment	N/A
Ethics oversight	N/A

Note that full information on the approval of the study protocol must also be provided in the manuscript.

Field-specific reporting

Please select the one below that is the best fit for your research. If you are not sure, read the appropriate sections before making your selection.

- [x] Life sciences
- [ ] Behavioural & social sciences
- [ ] Ecological, evolutionary & environmental sciences

For a reference copy of the document with all sections, see nature.com/documents/nr-reporting-summary-flat.pdf

Life sciences study design

All studies must disclose on these points even when the disclosure is negative.

Sample size	X-ray data were collected on single crystals until accepted completeness and redundancy were achieved. No sample size calculations were performed for the other experiments. Sample sizes were chosen based on previous experience with similar assays and accepted standards in the field, e.g., refs. 6, 23, 33, 70, 71.
Data exclusions	X-ray reflections were truncated based on CC1/2 and <(I)/(sigma I)>. No data were excluded from the biochemical analysis.
Replication	X-ray data were collected from single crystals. Biochemical experiments were performed with at least three replicates (unless otherwise stated), with all attempts at replication successful. Representative gels and graphs combining all three or more experiments with individual data points are shown for each experiment.
Randomization	5-10% of reflections for each diffraction dataset were randomly allocated by the CCP4 or Phenix programs from Rfree calculation. Randomization is not applicable to the other experiments as we compared different mutants and different mutant samples were processed in parallel.
Blinding	Blinding is not relevant to our study, since there are no experiments where investigator bias could affect measurements or data analysis. Band intensities were quantified using unbiased software.

Reporting for specific materials, systems and methods

We require information from authors about some types of materials, experimental systems and methods used in many studies. Here, indicate whether each material, system or method listed is relevant to your study. If you are not sure if a list item applies to your research, read the appropriate section before selecting a response.

Materials & experimental systems

n/a	Involved in the study
☒	☐ Antibodies
☒	☐ Eukaryotic cell lines
☒	☐ Palaeontology and archaeology
☒	☐ Animals and other organisms
☒	☐ Clinical data
☒	☐ Dual use research of concern

Methods

n/a	Involved in the study
☒	☐ ChIP-seq
☒	☐ Flow cytometry
☒	☐ MRI-based neuroimaging
